# Two-magnon bound state causes ultrafast thermally induced magnetisation switching

**DOI:** 10.1038/srep03262

**Published:** 2013-11-20

**Authors:** J. Barker, U. Atxitia, T. A. Ostler, O. Hovorka, O. Chubykalo-Fesenko, R. W. Chantrell

**Affiliations:** 1 Department of Physics, University of York, York YO10 5DD, U.K; 2 Departamento de Fisica de Materiales, Universidad del Pais Vasco, UPV/EHU, 20018 San Sebastian, Spain; 3 Instituto de Ciencia de Materiales de Madrid, CSIC, Cantoblanco, 28049 Madrid, Spain

## Abstract

There has been much interest recently in the discovery of thermally induced magnetisation switching using femtosecond laser excitation, where a ferrimagnetic system can be switched deterministically without an applied magnetic field. Experimental results suggest that the reversal occurs due to intrinsic material properties, but so far the microscopic mechanism responsible for reversal has not been identified. Using computational and analytic methods we show that the switching is caused by the excitation of two-magnon bound states, the properties of which are dependent on material factors. This discovery allows us to accurately predict the onset of switching and the identification of this mechanism will allow new classes of materials to be identified or designed for memory devices in the THz regime.

Thermally induced magnetisation switching (TIMS) occurs when an applied sub-picosecond heat pulse causes the magnetic state of a system to deterministically switch without any external or implicit magnetic field to determine the final state^1^. This unexpected switching was first observed in the amorphous rare earth-transition metal ferrimagnet GdFeCo and later, only in the similar materials TbCo^2^ and TbFe^3^, but always for a limited range of rare-earth concentration. As yet there is no explanation why this should be the case, nor what properties of the material allow TIMS to occur at all. Here we show that the microscopic origin of TIMS is the thermal excitation of two magnon bound states whose properties are dependent on the composition of the material and the exchange interactions present. Our results explain the plethora of somewhat contradictory and paradoxical experimental observations of all-optical switching (AOS), where each postulated material requirement for reversal has been countered by an opposing example in the literature^1^,^2^,^4^,^5^,^6^,^7^,^8^. This study focuses on the prototypical material GdFeCo, but our theory can be generalised, allowing the occurrence of TIMS to be predicted from a knowledge of the magnon band structure of a material. Our work gives the insight required to select materials or design heterostructures which can exploit TIMS for applications, such as all-optical magnetic storage, where the replacement of rare-earth materials is needed due to the issues surrounding the sourcing these elements.

In the area of all-optical switching, the explanation for switching magnetisation by laser light has shifted as more experiments are performed. In early papers it was thought that the inverse Faraday effect produced a large intrinsic magnetic field within GdFeCo which drove the magnetisation reversal^4^,^5^. More recent work by Khorsand *et al.* shows that the helicity dependence of AOS is due to magnetic circular dichroism causing a difference in the absorption of energy into the magnetic system^8^, supporting the results of Ref. 1. They show that there is a general threshold energy for AOS which is independent of the helicity. This result suggests that helicity dependent AOS is a subset of TIMS, where the magneto-optical properties of the material play a subsidiary role, but the underlying switching mechanism is purely magnetic. This is consistent with the discovery of TIMS where atomistic spin dynamics, which include no magneto-optical effects, predicted its existence prior to the experimental verification^1^.

Much has also been made of the role which the magnetisation or angular momentum compensation point plays in switching. AOS and TIMS are usually found close to these points and it has been suggested that heating across these points is a requirement for TIMS^2^,^7^. This explanation is contradicted by modelling and experiments which show switching without the traversal of these points^1^ and even in compounds with no compensation point^3^. A deeper understanding is therefore needed to explain these results in a consistent theory.

Several macroscopic and simple theoretical descriptions of the underlying physical mechanism have been proposed. These all express the switching as an exchange of angular momentum between magnetic sublattices, driven by the antiferromagnetic exchange coupling^9^,^10^. However, an accurate microscopic model of TIMS, validating these phenomenological descriptions has not yet been developed. A refined description would allow the quantification of both the intrinsic material properties and the control of the mechanism behind TIMS. This has been a key general research objective and is the focus of the present work.

Here we employ large-scale computational methods and rigorous analytic calculations to identify the microscopic origin of TIMS and demonstrate why switching is observed only for a limited range of rare-earth concentrations in the amorphous alloys introduced above. Our results are consistent with the experimental findings and we confirm the existence of a threshold energy required to induce switching. We find the threshold energy is related to the magnon band structure, therefore we can use the analytic framework of linear spin wave theory to make predictions about where this threshold is easy to overcome.

## Results

### GdFeCo random lattice model

As a prototypical material which exhibits TIMS, we construct a model of GdFeCo - a ferrimagnet where one species is attributed to Gd and the other species incorporates Fe and is an ‘effective moment’ of FeCo. The minimum effective Hamiltonian to capture the essential properties of the ferrimagnet is 
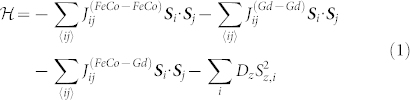
where ***S****_i_* = ***μ****_s,i_*/|***μ****_s,i_*|, ***μ****_s,i_* is the magnetic moment on lattice site *i*, 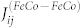, 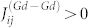 gives ferromagnetic ordering within each sublattice but 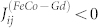 gives antiferromagnetic ordering between sublattices. *D_z_* is a small uniaxial anisotropy giving rise to an out of plane magnetization due to the small Co content (<10%). For the complete set of parameters see supplementary information table I. We also note that *μ_s,Gd_* > *μ_s,FeCo_* and 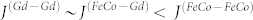, which ensures that the Gd demagnetisation is slower than that of Fe. These features are the common magnetic factors between the materials in which TIMS has been observed. Other effects such as magnetic changes in the magnetic anisotropy are expected to play a lesser role in the switching behaviour because TIMS is observed in both GdFeCo and TbCo where there is an order of magnitude difference in magnetic anisotropy.

**Figure 1:**
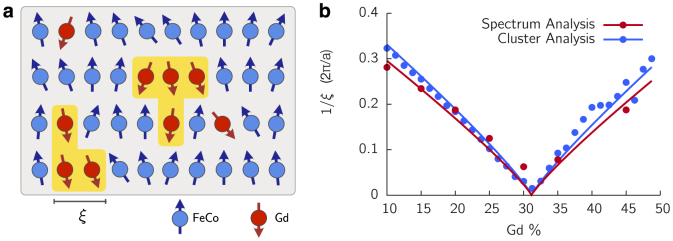
Clustering of Gd within FeCo lattice. (a) The lattice is made up of FeCo (blue) and Gd (red) spins. The random distribution of Gd within the lattice forms clusters of connected Gd regions. These have a typical length scale, *ξ*, for a given Gd concentration. (b) The cluster inverse correlation length (1/*ξ*), in terms of inverse length 2*π*/*a*, where *a* is the lattice constant, is found as a function of the Gd concentration using the Hoshen-Kopelman method (blue circles). The extent of the band overlap from the spin wave spectrum is also plotted (red circles). Both are fitted with a percolation theory 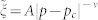.

The third important factor which we include is the amorphous nature of these alloys. TIMS has only been found for a limited range of rare-earth concentrations and we use this behaviour to gain insight into the microscopic mechanism. Therefore our model must be capable of representing off stoichiometric Gd*_x_*(FeCo)_1−*x*_ alloys. We model the amorphous nature of GdFeCo by randomising where Gd moments are placed within a large FeCo supercell (a simple cubic lattice of dimensions 128 × 128 × 128) until the desired concentration is achieved (Fig. 1a). Any randomly generated lattice such as this contains a statistical distribution of Gd cluster sizes, consistent with the percolation theory^11^ which gives the correlation length as 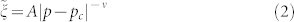
where *ν* = 0.875 is a critical exponent^12^, the percolation threshold is *p_c_* = 0.3116004 for a simple cubic lattice^13^ and *A* is a finite size scaling factor (Fig. 1b). The dynamics of the system are described by a set of coupled Landau-Lifshitz-Gilbert equations for each localized magnetic moment with a Gilbert damping of *α* = 0.02 which gives similar demagnetisation times to those observed in experiments^14^. To describe the thermal effect of the laser on the spin system, we couple the temperature in the LLG to the electronic temperature in the two-temperature model as defined in the Supplementary Information (S1).

### Microscopic dynamics during TIMS

The current theoretical and experimental understanding of TIMS is limited since it relies on the study of only macroscopic order parameters such as the total and individual sublattice magnetisations. To advance the understanding of TIMS and obtain a complete picture of the dynamics leading to switching, we first study the microscopic magnetisation dynamics. We do this by tracking the distribution of the spin fluctuations, magnons, during the reversal process through the calculation of the intermediate structure factor (ISF, see Supplementary Section S2), defined as 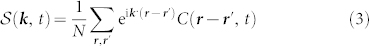
where N is the number of spins and *C*(***r*** − ***r***′, *t*) = 〈*S*_+_(***r***, *t*)*S*_−_(***r***′, *t*)〉 is the equal time spin-spin correlation function. The ISF tracks the magnon distribution within the Brillouin zone following the application of a femtosecond laser pulse.

**Figure 2:**
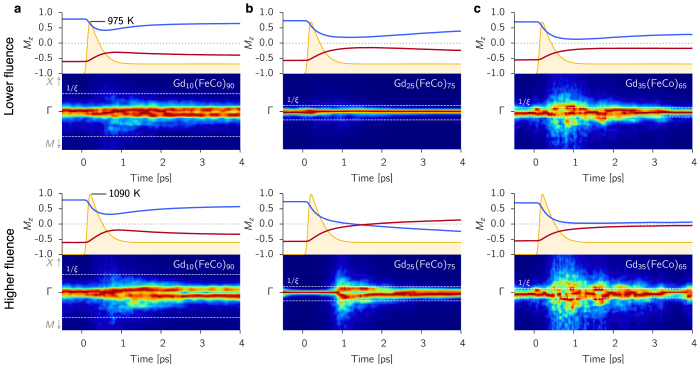
Intermediate structure factors with laser excitation. Magnetisation and intermediate structure factor dynamics after the application of the laser pulse to the amorphous lattice model. The blue and red lines are the *M_z_* of the FeCo and Gd, normalised to the total magnetisation of each sublattice respectively. The yellow curve shows the electronic temperature from the two-temperature model. In the lower panels the colour intensity represents the amplitude of magnons at the given *k*-vector normalised to the maximum value at that time. (a) Upper panel, the laser heating causes a reduction in the magnetisation and a redistribution of magnons in the Brillouin zone. Lower panel, increased heating causes more demagnetisation and magnons are excited at greater wave vectors, no switching occurs. (b) Upper panel, the laser heating causes a reduction in magnetisation of the two sublattices but the distribution of power in the magnons does not change significantly from the equilibrium distribution. Lower panel, a higher laser fluence causes switching. During the reversal period, magnons on a specific length scale are excited almost instantaneously, corresponding to the angular momentum transfer channel between AF and FM modes. After reversal the ISF returns to the equilibrium distribution. (c) Upper panel, the laser heating causes significant demagnetisation of both sublattices. In the ISF the power is broadly distributed with very short lived excitations at many different wave vectors. Lower panel, greater heating causes the same behaviour with a broader magnon distribution.

We find that three Gd concentration regimes can be defined with qualitatively different behaviour, specifically: low, switching and high regimes, where TIMS is found. The temporal behaviour of the ISF for each regime is shown in Fig. 2 as (a), (b) and (c) respectively.

#### Low Gd concentration - 10% Gd

The laser heating, represented by the yellow temperature profile (Fig. 2a), causes reduction of the magnetisation of the sublattices, leading to the emergent pattern in the ISF. This is due to thermally stimulated spin wave activity^15^. Increasing the laser fluence (lower figure) causes further demagnetisation but no switching. Thus, in this regime the dynamics are essentially those of a ferromagnet.

#### Switching Gd concentration ~ 20–30% Gd

TIMS occurs at this Gd concentration, although only above a threshold laser fluence (Fig. 2b). For laser fluences below the threshold (upper figure), the ISF shows that the absorbed laser energy is distributed within the low wave vector modes after the initial heating, leading to a decrease in the magnetisation of both sublattices. After the heating is removed, the electronic temperature equilibrates with the phonon temperature and the non-equilibrium magnon distribution returns towards equilibrium resulting in the gradual recovery in the magnetisation of the sublattices. For laser fluences above the switching threshold (lower figure) the initial heating leads to a more pronounced reduction in magnetisation, a basic requirement for TIMS^1^, and the excitation of a broader *k*-range. While cooling, instead of a relaxation of magnons, the instantaneous excitation of magnons is observed within a well defined range in *k*-space. From the macroscopic magnetisation point of view, this initially leads to the so-called transient ferromagnetic-like state^1^ which precedes TIMS. The length scale on which this occurs is commensurate with the characteristic length scale of Gd clusters in the lattice (shown as 1/*ξ*).

#### High Gd concentration - 35% Gd

The laser heating has a more significant effect due to the reduction in *T_c_* with increasing Gd content. The ISF shows that regions of non-zero *k* with very short life times are excited. For a high laser fluence (lower figure) the lattice is almost completely demagnetised by these short lived excitations. We note that although the condition of complete FeCo sublattice demagnetisation is fulfilled, TIMS does not take place, suggesting that the reversal mechanism is ineffective for this composition.

To identify the nature of magnons defining the angular momentum transfer channels, we calculate the equilibrium dynamic structure factor (DSF), 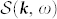, to obtain the magnon spectrum (experimentally obtainable via Brillouin light scattering experiments). In a pure ferromagnet one would observe a single band corresponding to one magnon excitations which describes the frequency-wave vector dependence of the thermally excited magnons. In magnetic materials with multiple species, a band per species occurs. Thus, the amplitude of bands on the same wave vector, 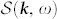, conveys information about one- and two-magnon band contributions. To give a clear contrast of the relative contribution to the spin fluctuations of each magnon branch in the spectrum we perform the normalisation 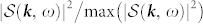 at each ***k*** (Ref. 16). Finally, to interpret the ISF in relation to the equilibrium spectrum, we assume the laser heating is so fast that only the population of magnons within the spectrum is altered (according to the colour scheme in Fig. 2) but the spectrum, *ω* vs. ***k***, is not significantly altered in itself.

The spectrum in ferrimagnetic GdFeCo alloys contains two magnonic bands where the low energy spectrum, *ω*(*k*), of both branches contains a mixture of linear and quadratic behaviour in *k*. Based on the predominant character of each band in the low and high Gd concentration limits respective, we define 1) the FM-like band whose low-energy scales *ω*(*k*) ~ *k*^2^ (see Fig. 1c) and 2) the AF-like band where the low energy magnon dispersion is *ω*(*k*) ~ *k*. The interaction between the sublattices gives rise to a band gap at *k* = 0 due to the exchange field between the lattices which follows from Δ*f*_0_ ~ *J_AB_*(*M_A_* − *M_B_*) and therefore the gap depends on the relative concentration of each species within the lattice. The magnon branches and the band gap evolve in a characteristic manner with increasing Gd concentration.

**Figure 3:**
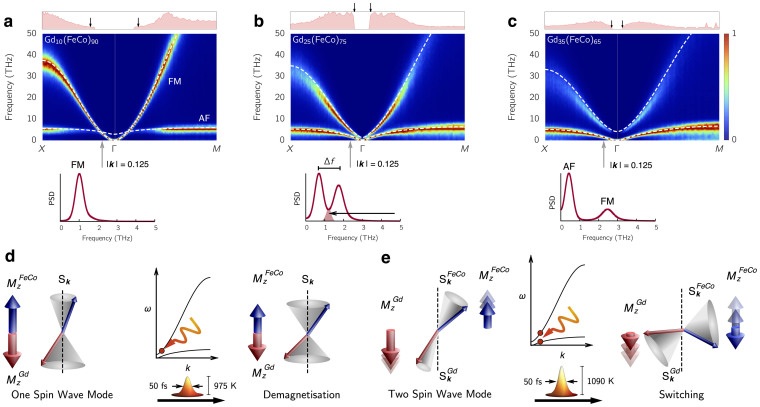
Magnon band structures and explanation of one and two-magnon states. (a–c) In each DSF the colour indicates the relative amplitude of magnon modes (the power spectrum density) normalised by the maximum value on each wave vector. The analytic dispersion from the LSWT is overlaid on each DSF in dashed-white, showing a good agreement with our calculated band structure. The box above each panel gives the amplitude of the two-magnon state in red. This is maximised when both bands have the same amplitude and is zero where only one band contains any amplitude. The mean Gd cluster correlation length *ξ* as calculated from percolation theory is denoted by the black arrows. Below each DSF is a cross section of the spectrum at the *k* vector |*k*| = 0.125. (a) Low Gd concentration has distinct FM and AF magnons. The AF band is restricted to the edge of the Brillouin zone (coloring scheme) as there are relatively few, localised FeCo-Gd interactions. The system behaves as a FM due to the dominance of this band. (b) For Gd ~ 20–30%. there is region near the centre of the Brillouin zone with the two-magnon state and a small frequency gap (Δ*f*) between the two bands. The shaded region in the PSD is where non-linear interactions allow the efficient transfer of angular momentum between sublattices. Strong excitation of these magnons causes TIMS. (c) High Gd concentration reduces the two-magnon state and the large frequency gap stops the flow of angular momentum between the FM and AF modes. (d) Excitation of one magnon modes causes a reduction in the magnetisation. (e) Two-magnon modes cause localised oscillations in the magnetisation. Strong excitation of these states causes a transfer of angular momentum between FM and AF modes leading to the transient ferromagnetic state and switching.

At low Gd concentrations, where TIMS is not observed in our simulations nor in experiments^17^, Gd can be considered an isolated impurity, rather than a cluster, in the FeCo lattice and the distinction between FM and AF magnons is well manifested (Fig. 3a). The spectrum is dominated mainly by the FM spin fluctuations and the FM mode is dominant across the Brillouin zone. It is only at the edge of the Brillouin zone where the few localized FeCo-Gd interactions cause the AF mode and the FM mode to be similar in amplitude. This indicates the suppression of AF excitations on longer length scales (small k) within the lattice and thus the interaction-induced AF mode is range-limited. The colour scheme in Fig. 3 indicates the relative intensity of the bands, not the absolute intensity, which suggests that in the low-energy regime GdFeCo essentially behaves as a ferromagnet slightly perturbed by Gd impurities. The modes are mixed only at very short correlated length scales (large *k* values) which are not accessible to femtosecond laser heating, so the laser heating excites only FM modes leading to a reduction in magnetisation (Fig. 3d).

As the Gd concentration increases, so does the FeCo-Gd AF exchange interaction correlation length, and both FM and AF magnons have a similar relative amplitude on the same length scales. Consequently, in the FeCo lattice the relative FM magnon contribution to spin fluctuations decreases in amplitude at large length scales in favour of the AF modes (Fig. 3b), gradually diminishing the ferromagnetic character of such spin fluctuations to a FM-AF magnon mixing - the two-magnon bound state. For 20–30% Gd, there exists a strong interplay between the two bands and a region develops close to the centre of the Brillouin zone where the relative amplitude of both magnon branches is similar, leading to localised oscillations in the magnetisation vector that can be excited by the laser energy as shown in figure 3e. This is a key factor, allowing angular momentum transfer between the modes which scales with the intersecting area of the two bands (see cross section below Fig. 3b). This area is maximised when the gap between the bands, Δ*f* is minimised. For TIMS to occur the transfer of angular momentum from FM to AF modes must be enhanced. This occurs for increasing laser fluence because the number of thermally excited magnons involved in angular momentum transfer is increased.

For even larger Gd content (>35%) FeCo-Gd interactions play the dominant role in the lattice. The system takes on the character of an antiferromagnet with negligible contribution from the FM band which is now raised in frequency due to the increased coupling strength between Gd and Fe sublattices, proportional to Gd concentration (see supplementary information S4) (Fig. 3c). The large frequency gap Δ*f* means that there are negligible interactions between two-magnon modes, reducing angular momentum transfer, and supplying the laser energy causes the system to demagnetise via the excitation of one magnon branch, as in figure 3d.

In the panels above each DSF we measure the amplitude of two magnons at a given *k* corresponding to FM and AF branches (red filled curves which relates to the colouring scheme in the main plot). When this is maximised, the relative (normalised) amplitude of both magnon branches is the same, zero indicates the presence of only one branch on that wave vector. We compare the largest length scale on which two-magnon modes occur, with the typical length scale of Gd cluster in the lattice *ξ* (black arrows in Fig. 3). We make a more extensive comparison in Fig. 1b where the cluster analysis (of Gd lattice sites) is performed using the Hoshen-Kopelman method (see Methods) and the spectrum analysis is taken from the two-magnon amplitude in the DSF. The length scale of the two-magnon states and the Gd lattice clusters is almost identical, indicating that the FM-AF interfaces of the rare-earth clusters play a role. With this knowledge we can predict the extent of the two-magnon bound state of the magnon spectrum.

### Predicting TIMS

The minimum laser energy required to initiate switching is essential in the interpretation of experiments^8^,^18^. From our understanding of the microscopic origin of TIMS we propose the following criterion for the intrinsic material properties. First, the frequency gap Δ*f*(*k*) between both magnon branches should be minimised in order to maximise the angular momentum transfer through nonlinear interactions (Fig. 3b). Secondly, the laser energy must be sufficient to strongly excite the two-magnon bound states where they occur in the Brillouin zone. The relationship between the Gd clusters and the wave vector allows us to determine the length scale on which FM and AF modes have the largest mixing from Eq. 2. We plot 1/*ξ* as dashed white lines in Fig. 2 where it matches the excited region during switching and as black arrows in the top panels in Fig. 3a–c where it matches the extent of the two-magnon state in the DSF, shown in red above each panel.

**Figure 4:**
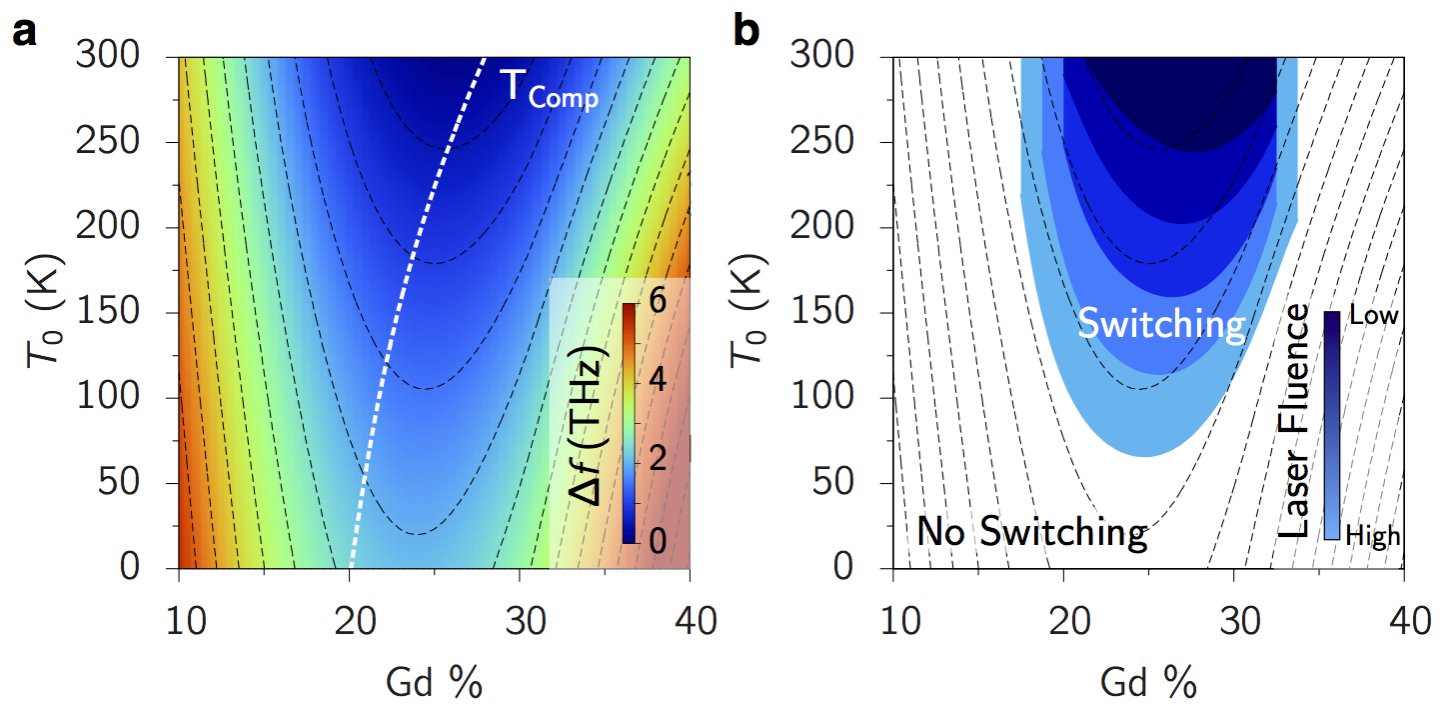
TIMS reversal windows. (a) The band frequency difference Δ*f* at the cluster correlation length *ξ*, calculated from LSWT with the VCA and percolation theory. The spacial localisation of the Gd clusters means the minimum does not lie on *M_comp_*. Smaller Δ*f* means that the two-magnon modes can more efficiently transfer angular momentum and magnetisation between sublattices when sufficiently excited. (b) TIMS switching windows found from atomistic spin dynamics for different laser fluence. *T*_0_ is the initial temperature and the thermal evolution is calculated from the two-temperature model where the peak electronic temperature depends on the laser fluence. Each enclosed area is the parameter set where switching occurs for a constant, labelled laser fluence (in J/sm^3^), effectively a constant energy input. The switching windows closely match the energy contours from diagram (a) (shown in grey).

The magnon spectrum can be calculated from linear spin wave theory (LSWT) where we use the virtual crystal approximation (VCA) to simplify the amorphous character of our spin model (see Supplementary Information S4). The resulting spectrum is shown as a dashed white line over our DSF calculations in Fig. 3, validating the VCA. The theory also demonstrates the mixing of FM and AF magnons through the transformation of the equations of motion (see Supplementary Information S4) in agreement with numerical calculations. From our analytic framework and using *ξ* calculated via percolation theory, we calculate Δ*f*(1/*ξ*), the frequency difference of the low energy two-magnon states, for the temperature range *T* = 0–300 K and Gd concentrations, 10%–40%, i.e. an experimentally accessible parameter space where the temperature is the initial temperature *T*_0_ before the heat pulse (Fig. 4a). Within the adiabatic approximation it is the spectrum calculated at *T*_0_ within which the magnons will be redistributed by the heating.

To test our premiss that the threshold laser energy to induce TIMS scales with Δ*f*(1/*ξ*), we perform extensive computational simulations as in Fig. 2 to find the regions of this parameter space where TIMS occurs. Fig. 4b) shows the switching regions for several different laser fluences. The parameters at which Δ*f*(1/*ξ*) is smallest are around Gd concentrations of 25%, but the minimum for any given temperature does not coincide with the magnetisation compensation point, *M*_comp_, due to the excitation at non-zero *k*-vector. This deviation directly relates to the Gd clustering which limits the range of the two-magnon states. In larger samples the FeCo clustering around Gd rich regions can produce the inverse effect, namely, showing a transfer of angular momentum to FeCo clusters with the consequence of Gd region reversing first^19^. If the two-magnon states were to exist at the Γ-point, then the minimum of Δ*f*(1/*ξ*) does in fact follow *M*_comp_ and thus switching is easiest at the compensation point in this case.

## Discussion

Our study has identified the nature of TIMS as the excitation of two-magnon bound states where the energy is transfered from FM to AF modes via non-linear interactions. This mediates the angular momentum transfer between ferromagnetic subsystems with antiferromagnetic coupling between them through the AF interactions at the cluster interfaces. Our quantitative analysis opens the door for design of magnetic heterostructures for more energy-efficient all-optical storage devices^20^, and an enhancement of the information processing rates into the elusive THz regime^5^. The angular momentum transfer channels identified in this work as being essential for the occurrence of TIMS, can be directly accessed by THz excitation by a magnetic or electric field^21^,^22^,^23^. Operation in the THz range leads to a range of benefits as it substantially reduces the heat generation that leads to material fatigue and device performance degradation. Additionally, due to the problem with sourcing the rare-earth materials, the large-scale technological impact relies on the discovery of new cost-friendly TIMS-exhibiting materials. As suggested in this work, the relatively small parameter space necessary for existence of TIMS in natural materials such as the GdFeCo alloys can be broadened via engineering of heterostructures, for instance, superlattices made of ferromagnetic layers with strong AF coupling, and by improving the inter-lattice magnon-exchange efficiency. The framework we have laid out here applies equally to such regular structures, with the exception of the amorphous clustering. For example in a layered structure the spatial correlation length would exist across the entire film, thus it is the band gap at the Γ-point which is relevant. According to the LSWT this band gap is always minimised at *M*_comp_ thus there may be significant advantages, such as a lower threshold energy, for TIMS in such artificial materials.

## Methods

### Atomistic spin model

The laser heating is modelled using a two-temperature model representing the coupled phonon and electron heat baths. The spin degrees of freedom are coupled to the electronic temperature. Calculation of the dynamic structure factor was by means of a three dimensional spacial discrete Fourier transform (with periodic boundaries) and temporal discrete Fourier transform where a Hamming window is applied. We use a simple cubic lattice of size 128 × 128 × 128 and integrate the coupled Landau-Lifshitz-Gilbert Langevin equations for over 800 ps of simulated time, giving a frequency resolution of 2.5 GHz. The resulting power spectra are then convoluted along constant *k*-vector with a Gaussian kernel of width ~ 0.95 THz and normalised so the largest peak is unity (an example is given in Supplementary Fig. 1).

### Linear spin wave theory

We first use the virtual crystal approximation to make the disordered lattice Hamiltonian in Eq. (1) translationally symmetric with respect to spin variables. The magnon spectrum is described by the linearized Landau-Lifshitz equation of motion, d**s***_i_*/d*t* = *γ*[**s***_i_* × **H**_eff,*i*_], where 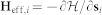. The resulting equations are then transformed in terms of spin raising and lowering operators 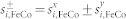 and 
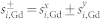
 which describe the spin fluctuations around equilibrium. The resulting system of two coupled equations is then Fourier transformed to describe the spin fluctuations in the reciprocal space and diagonalised by a Bogoliubov-like transformation 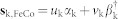, 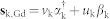, where *α*_k_ and *β*_k_ are the eigenstates (magnons) of the system with frequency *ω_α_*(**k**) and *ω_β_*(**k**) respectively. The coefficients *u*_k_, *v*_k_ and more detail is given Supplementary Section S4.

### Percolation theory

Percolation theory provides a general mathematical toolbox for quantifying statistical properties of connected geometrical regions of size *s* which will here refer to *s* adjacent Gd atom sites. After identifying such Gd clusters within the lattice using the efficient Hoshen-Kopelman algorithm^24^, discounting small clusters (*s* < 4) and percolating clusters spanning the computational cell, we calculate the radius of gyration *R_st_* of each cluster remaining within the distribution and obtain the correlation length as: 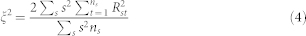
The finite size effects are included via the scaling formula for the correlation length 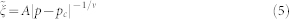
where *p_c_* is the percolation threshold for bulk lattice and *ν* is correlation length universal critical exponents. The values *p_c_* = 0.3116004 and *ν* = 0.875 for site percolation on a simple cubic lattice and the non-universal constant *A* = 0.776187 obtained by fitting Eq. 3 to the cluster data evaluated by statistical counts through the lattice. Thus Eq. 3 allows relating the Gd concentration to the associated typical geometrical size of Gd clusters, and correlates well with the predictions of the LSWT discussed in the text.

## Author Contributions

J.B. and T.O. performed atomistic spin dynamics simulations; J.B., U.A. and O.F. performed the LSWT calculations and interpretation and J.B., O.H. and R.C. carried out the HK cluster analysis and percolation theory. J.B. and U.A. wrote the core of the manuscript, all authors contributed to parts of it.

## Supplementary Material

Supplementary Information Supplementary Information
