# Supplementary material for: Two-magnon bound state causes ultrafast thermally induced magnetisation switching
Source: Sci Rep. 2013 Nov 20;3:3262. doi: 10.1038/srep03262 (PMC3834892; doi:10.1038/srep03262)
Supplement: Supplementary Information [file srep03262-s1.pdf]

Two-magnon bound state causes ultrafast thermally induced magnetisation switching Supplementary Information

J. Barker*,[1] U. Atxitia,[1,2] T.A. Ostler,[1] O. Hovorka,[1] O. Chubykalo-Fesenko,[3] and R.W. Chantrell[1]

[1]*Department of Physics, University of York, York YO10 5DD, U.K.*
[2]*Departamento de Fisica de Materiales, Universidad del Pais Vasco, UPV/EHU, 20018 San Sebastian, Spain*
[3]*Instituto de Ciencia de Materiales de Madrid, CSIC, Cantoblanco, 28049 Madrid, Spain.*

S1 - ATOMISTIC SPIN MODEL

The atomistic modelling used in this work follows standard techniques in this area. We include a description of the model here for completeness. We use the Landau-Lifshitz-Gilbert equation

$$\frac{\partial \boldsymbol{S}_i}{\partial t} = -\frac{\gamma_i}{(1+\alpha_i^2)\mu_i}\left(\boldsymbol{S}_i \times \boldsymbol{H}_i + \alpha_i \boldsymbol{S}_i \times \boldsymbol{S}_i \times \boldsymbol{H}_i\right) \tag{1}$$

where γ_i is the gyromagnetic ratio, α_i is the Gilbert damping, μ_i is the magnetic moment and $\boldsymbol{H}_i$ is the effective field on a spin $\boldsymbol{S}_i$. We can include temperature by writing the LLG as a Langevin equation, where the effective field contains a stochastic process $\boldsymbol{\xi}_i$

$$\boldsymbol{H}_i = \boldsymbol{\xi}_i - \frac{\partial \mathcal{H}}{\partial \boldsymbol{S}_i} \tag{2}$$

the moments of which are defined as

$$\begin{aligned} \langle \xi_i(t) \rangle &= 0 \\ \langle \xi_{i,a}(t), \xi_{j,b}(t') \rangle &= (2k_B T \alpha_i \mu_i/\gamma_i)\delta(|t-t'|)\delta_{ij}\delta_{ab} \end{aligned} \tag{3}$$

where a and b are Cartesian components. The equation of motion is integrated with the Heun scheme using a time step of dt=0.1 fs to ensure numerical stability. The material parameters we use in the model are given in table I. The amorphous nature of GdFeCo is modelled by using a simple cubic lattice model but with random

FeCo-FeCo Exchange Energy	J_{ij}	6.920×10^{-21}	(J)
FeCo-Gd Exchange Energy	J_{ij}	-2.410×10^{-21}	(J)
Gd-Gd Exchange Energy	J_{ij}	2.778×10^{-21}	(J)
FeCo Anisotropy Energy	d_z	8.072×10^{-24}	(J)
FeCo Moment	μ_s	1.92	(μ_B)
FeCo Damping	α	0.02	
FeCo Gyromagnetic Ratio	γ	1.00	(γ_e)
Gd Anisotropy Energy	d_z	8.072×10^{-24}	(J)
Gd Moment	μ_s	7.63	(μ_B)
Gd Damping	α	0.02	
Gd Gyromagnetic Ratio	γ	1.00	(γ_e)

TABLE I: Atomistic material parameters for GdFeCo in the LLG equation.

placements of Gd moments within the lattice to obtain a specific concentration. Using a very large lattice ($128 \times 128 \times 128$) allows use to finely control the concentration and also gives a good ensemble of clusters.

The thermal effect of the laser is included by use of the two temperature model where the spin system is coupled to the electron temperature[1,2]. The two temperature model is written

$$\begin{aligned} C_e\frac{dT_e}{dt} &= -G(T_e - T_p) + P(t) \\ C_p\frac{dT_p}{dt} &= G(T_e - T_p) \end{aligned} \tag{4}$$

where T_p is the phonon heat bath temperature, T_e is the electronic heat bath temperature to which we couple the LLG equation of motion. $P(t)$ is the laser power which we include as a Gaussian, $P(t) = \mathcal{P}\exp(-(t-3t_p)^2/t_p^2)$ with a pump time of $t_p = 50$fs and $\mathcal{P}$ is the power density, or fluence in units of $\mathrm{Js^{-1}m^{-3}}$. $G = 1.7 \times 10^{18}$ J/sKm^3 is the electron phonon coupling strength. $C_e = \gamma T_e$, $\gamma = 700$ J/Km^3 and $C_p = 3 \times 10^6$ J/Km^3 are the electronic and phonon specific heat capacities respectively.

S2 - STRUCTURE FACTORS

The intermediate structure factor (ISF) is calculated from

$$\mathcal{S}(\boldsymbol{k}, t) = \frac{1}{N}\sum_{\boldsymbol{r},\boldsymbol{r}'} \mathrm{e}^{\mathrm{i}\boldsymbol{k}\cdot(\boldsymbol{r}-\boldsymbol{r}')} C\left(\boldsymbol{r}-\boldsymbol{r}', t\right) \tag{5}$$

where N is the number of spins and the spin-spin correlation function, $C(\boldsymbol{r}-\boldsymbol{r}', t) = \langle S_+(\boldsymbol{r}, t)S_-(\boldsymbol{r}', t)\rangle$. The dynamic structure factor (DSF) is calculated from

$$\mathcal{S}\left(\boldsymbol{k}, \omega\right) = \frac{1}{N}\sum_{\boldsymbol{r},\boldsymbol{r}'} \mathrm{e}^{\mathrm{i}\boldsymbol{k}\cdot(\boldsymbol{r}-\boldsymbol{r}')} \int \mathrm{e}^{\mathrm{i}\omega t} C\left(\boldsymbol{r}-\boldsymbol{r}', t\right) \mathrm{d}t \tag{6}$$

where N is the number of spins, $C(\boldsymbol{r}-\boldsymbol{r}', t) = \langle S_+(\boldsymbol{r}, t)S_-(\boldsymbol{r}', 0)\rangle$ is the spin-spin correlation function. S_+, S_- are the spin raising and lowing operators and $\langle\cdots\rangle$ denotes a thermodynamic average. Numerically the time integral is performed as a discrete Fourier transform in a Hamming window.

To reduce noise and improve the readability of the structure factors, the data is processed following the same techniques used by Bergman *etal.*[3]. For the DSF, the data on each constant k-vector, the data is first smoothed with a Gaussian convolution of width 0.95 THz and then normalised so that the maximum value is unity. This means that the mode amplitudes can be compared only on constant k-vectors but not between k-vectors. In the ISF the smoothing is applied along constant time. The normalisation is useful, especially when looking at the intermediate structure factors because otherwise the large change in temperature across the ISF would make the difference in contrast between low and high temperature too large to reasonably display. Further more we do no wish to compare the absolute value of the amplitude at different times, but rather see which modes are more populated at each given instance in time. An example of the Gaussian convolution is given in figure 1 showing that the data is smoothed but the features remain intact.

S3 - CLUSTER COUNTING AND PERCOLATION THEORY

To identify clusters of Gd sites in the lattice we use the Hoshen-Kopelman method[4]. This is an efficient algorithm for identifying unique clusters on a lattice. We define a unique cluster as any set of Gd sites which are linked together by a immediate adjacent site (i.e. nearest neighbour exchange coupled). To calculate the typical correlation length, we first remove the tails of the distribution[5], that is any cluster of size $s < 4$ and the percolating cluster. In practice we discount the single largest cluster to avoid having to calculate if a cluster is

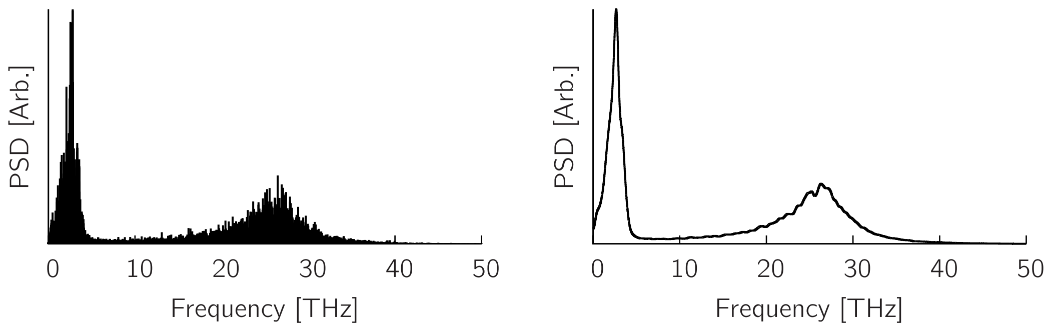

(a) Raw PSD data

(b) PSD data after Gaussian convolution

FIG. 1: The power spectral density along one k-vector is filtered using Gaussian convolution with a width of $\sigma = 0.95$ THz.

percolating. For the calculation of the correlation length, ξ we use the formula[5]

$$\xi^2 = \frac{2\sum_s s^2 \sum_{t=1}^{n_s} R_{st}^2}{\sum_s s^2 n_s} \tag{7}$$

where s is the cluster size, R is the radius of gyration of a cluster and n_s is the number of clusters of size s. Universal critical exponents are strictly speaking defined only in the thermodynamic limit. Their determination from finite size lattice simulations requires performing finite size scaling analysis[5]. However, considering very large lattices in our simulations reduces finite size effects and allows direct determination of critical exponents by fitting to the lattices we generate using the form

$$\tilde{\xi} = A|p - p_c|^{-\nu} \tag{8}$$

where p_c is the site percolation threshold which is 0.3116004 for a simple cubic lattice[6] and the critical exponent ν is 0.875 (ref. 7) and A is the only fitted parameter. The resulting fit is shown in figure 2 along with data extracted directly from the DSF as the maximum length scale of the two-spin region. These show a good agreement as discussed in the article.

S4 - FERRIMAGNET LINEAR SPIN WAVE THEORY

The magnon spectra of a disordered ferrimagnetic spin lattice is calculated analytically within the framework of the linear spin-wave theory by linearising the Landau-Lifshitz equation of motion. To calculate the magnon spectra for arbitrary Gd composition it is needed to transform the Heisenberg Hamiltonian (see Methods section of main article) from the non-translationally symmetric form (with respect to spin variables $\mathbf{s}_i$), to a symmetric one. This is achieved using the spin analogy of the virtual crystal approximation (VCA) to transform the disordered lattice Hamiltonian $\mathcal{H}$ to a symmetric VCA Hamiltonian $\mathcal{H}_{\mathrm{VCA}}$. Within the VCA we evaluate the effective sublattice exchange parameters, given by the sum of the exchange interactions of a given spin at a site $\mathbf{r}_i$ of sublattice i with all other atoms of this sublattice. This involves weighting the exchange parameters by the relative composition, $x_i \equiv$ concentration species i,

$$J_i = \sum_{\mathrm{r}_i, \mathrm{r}_i'} J(\mathbf{r}_i, \mathbf{r}_i') \underbrace{\equiv}_{\mathrm{VCA}} x_i J(\mathbf{r}_i, \mathbf{r}_i') \quad \text{intrasublattice} \tag{9}$$

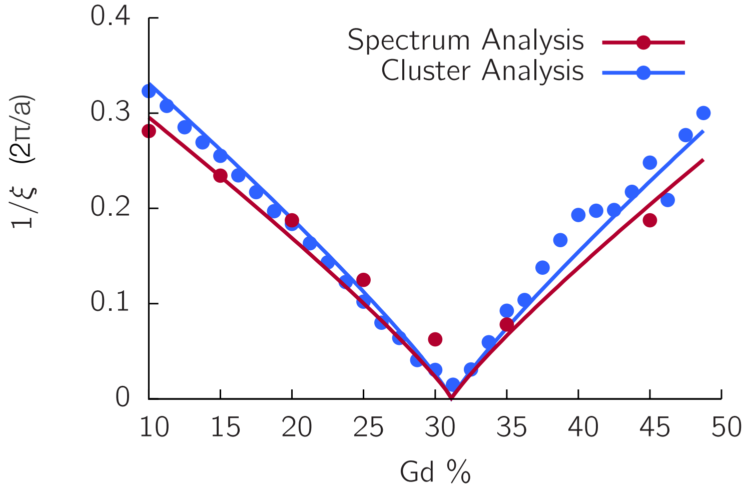

FIG. 2: The red points correspond to data taken from cluster analysis using the Hoshen-Kopelman method on the lattice. The blue points are the maximum extent of the two spin wave mode as measured from the Langevin-Landau-Lifshitz-Gilbert dynamic structure factors (see Figure 2 in the main article). The lines correspond to the fit of Eq. (8) where only A is a free parameter. For the cluster analysis $A = 0.776187 \pm 0.01142$, analysis from the DSF gives $A = 0.869468 \pm 0.04154$.

whereas the intersublattice effective exchange reads

$$J_{ij} = \sum_{\mathrm{r}_i, \mathrm{r}'_j \notin A_i} J(\mathbf{r}_i, \mathbf{r}'_j) \underbrace{\equiv}_{\mathrm{VCA}} x_i J(\mathbf{r}_i, \mathbf{r}'_j) \quad \text{intersublattice} \tag{10}$$

Thus the VCA Hamiltonian reads

$$\mathcal{H}_{\mathrm{VCA}} = \sum_{j \in A_i} J_i \mathbf{S}_i \cdot \mathbf{S}_j + \sum_{j \notin A_i} J_{ij} \mathbf{S}_i \cdot \mathbf{S}_j \tag{11}$$

where A_i represents the magnetic sublattice of the spin $\mathbf{S}_i$. The Fourier transform of the LL equation describes the spin fluctuations in the reciprocal space. The dynamical equation in terms of spin raising and lowering operators $S_i^{\pm} = S_i^x \pm S_i^y$ is written with

$$\mathrm{d}_t \begin{pmatrix} S_1^+(\mathbf{k}) \\ S_2^-(\mathbf{k}) \end{pmatrix} = -i \begin{pmatrix} \Omega_1(\mathbf{k}) + \frac{\gamma}{\mu_1} m_2 J_{12}(0) & \frac{\gamma}{\mu_1} m_2 J_{12}(\mathbf{k}) \\ \frac{\gamma}{\mu_2} m_1 J_{21}(\mathbf{k}) & \Omega_2(\mathbf{k}) + \frac{\gamma}{\mu_2} m_1 J_{21}(0) \end{pmatrix} \begin{pmatrix} S_1^+(\mathbf{k}) \\ S_2^-(\mathbf{k}) \end{pmatrix} \tag{12}$$

where we have defined $\Omega_n(\mathbf{k}) = \frac{\gamma}{\mu_n} m_n (J_n(0) - J_n(\mathbf{k}))$ where $J_n(\mathbf{k}) = \sum_{\mathrm{R}} J_n(\mathbf{R}) \exp(-i\mathbf{k} \cdot \mathbf{R})$ and $J_{nm}(\mathbf{k}) = \sum_{\mathrm{R}} J_{nm}(\mathbf{R}) \exp(-i\mathbf{k} \cdot \mathbf{R})$. We note that $\Omega_n(\mathbf{k})$ corresponds to the dispersion relation of the individual sublattice ferromagnetic magnons that would exist in each lattice in the absence of inter lattice exchange interaction. We include the effect of the temperature in $\mathcal{H}_{\mathrm{VCA}}$ (11) by the assumption of $|\langle \mathbf{S}_i(T) \rangle| = m_i(T)$, where m_i is the temperature dependent magnetization of the sublattice i normalized as $m_i = M_i(T)/M_i(T = 0\ \mathrm{K})$. The values of m_1 and m_2 can be calculated in the mean-field approximation (MFA) by solving the self-consistent Curie-Weiss equations $m_{e,1}(T) = L(\beta J_{\mathrm{ex},1})$ and $m_{e,2}(T) = L(\beta J_{\mathrm{ex},2})$, where L is the Langevin function and

$\beta = 1/(k_B T)$ (See Ref. 8). The mean exchange on each sublattice is given by

$$
\begin{aligned}
J_{\text{ex},1} &= J_{0,11} m_1 + J_{0,12} m_2 \\
J_{\text{ex},2} &= J_{0,22} m_2 + J_{0,21} m_1
\end{aligned} \tag{13}
$$

and

$$
\begin{aligned}
J_{0,11} &= (1-x) z J_{11} \\
J_{0,22} &= x z J_{22} \\
J_{0,12} &= x z J_{12} \\
J_{0,21} &= (1-x) z J_{21}
\end{aligned}
$$

where subscripts 1 and 2 denote two different species, x is the concentration of species 2, z is the coordination of the lattice.

The matricial equation (12) is diagonalised so that the two eigenvectors (magnons) can be written as a linear combination of spin lowering and raising operators as follows;

$$
\begin{aligned}
\alpha_{\text{k}} &= u_{\text{k}} S^{+}_{\text{k},1} + v_{\text{k}} S^{-}_{\text{k},2} \\
\beta^{\dagger}_{\text{k}} &= v_{\text{k}} S^{+}_{\text{k},1} + u_{\text{k}} S^{-}_{\text{k},2}
\end{aligned} \tag{14}
$$

The coefficients u_{k} and v_{k} are the eigen-vectors coefficients. We note that we are using the dynamical LL equation approach to calculate the frequency of the normal oscillations for classical spin operators. The same results are obtained by using the Holstein-Primakov approach to linearise the VCA Hamiltonian. The corresponding eigenvalues (energy of magnons) are

$$
\omega_{\alpha}(\mathbf{k}) = \Omega_{-}(\mathbf{k}) + \Omega^{-}_{12} + \sqrt{\left(\Omega_{+}(\mathbf{k}) + \Omega^{+}_{12}\right)^2 - C(\mathbf{k})} \tag{15}
$$

$$
\omega_{\beta}(\mathbf{k}) = -(\Omega_{-}(\mathbf{k}) + \Omega^{-}_{12}) + \sqrt{\left(\Omega_{+}(\mathbf{k}) + \Omega^{+}_{12}\right)^2 - C(\mathbf{k})} \tag{16}
$$

$M_n = x_n \mu_n m_n$ and $A_{12} = z|J(\boldsymbol{r}_1, \boldsymbol{r}_2)|/(\mu_1 \mu_2)$ are the macroscopic individual magnetization and the inter-lattice micromagnetic exchange parameter. We define $C(\mathbf{k}) = (2\gamma A_{12})^2 M_1 M_2$, we also define $2\Omega_{\pm}(\mathbf{k}) = \Omega_1(\mathbf{k}) \pm \Omega_2(\mathbf{k})$ and $2\Omega^{\pm}_{12} = \gamma A_{12}(M_2 \pm M_1)$. The eigenvector coefficients $(u_{\text{k}}, v_{\text{k}})$ can be calculated as

$$
u^2_{\text{k}} = \frac{1}{2}\left(\frac{\Omega_{+}(\mathbf{k}) + \Omega^{+}_{12}}{\sqrt{\left(\Omega_{+}(\mathbf{k}) + \Omega^{+}_{12}\right)^2 - C(\mathbf{k})}} + 1\right), \quad v^2_{\text{k}} = \frac{1}{2}\left(\frac{\Omega_{+}(\mathbf{k}) + \Omega^{+}_{12}}{\sqrt{\left(\Omega_{+}(\mathbf{k}) + \Omega^{+}_{12}\right)^2 - C(\mathbf{k})}} - 1\right). \tag{17}
$$

And

$$
2 u_{\text{k}} v_{\text{k}} = \left(\frac{C(\mathbf{k})}{\sqrt{\left(\Omega_{+}(\mathbf{k}) + \Omega^{+}_{12}\right)^2 - C(\mathbf{k})}}\right)^{1/2} \tag{18}
$$

Here we note that at $\mathbf{k} = 0$ a gap between magnon branches is opened, $\Delta\omega(0) = \omega_{\alpha}(0) - \omega_{\beta}(0) = 2\Omega^{-}_{12} = \gamma A_{12}(M_2(T) - M_1(T))$ which tends to minimise approaching the compensation temperature, where $M_1(T_M) = M_2(T_M)$ this gap disappears. Differently to ferromagnets, where the gap relates to the energy needed to make precessing spin fluctuations around the anisotropy and applied field, in ferrimagnets, the appearance of a gap at $\mathbf{k} = 0$, $\Delta\omega(0)$, is related to the minimum energy needed to precess each lattice spin fluctuations around the exchange field generated by the other sublattice.

The spontaneous magnetization of the system $\langle S^z \rangle = \langle S_1^z \rangle + \langle S_2^z \rangle$ is the sum of magnetization on the two sublattices. In terms of of the lowering and raising spin operators it can be written

$$\langle S_1^z \rangle = 1 - \frac{1}{\mathcal{N}_1} \sum_{\mathrm{k} \in B_z} \langle s_{\mathrm{k},1}^+ s_{\mathrm{k},1}^- \rangle \tag{19}$$

$$\langle S_2^z \rangle = -1 + \frac{1}{\mathcal{N}_2} \sum_{\mathrm{k} \in B_z} \langle s_{\mathrm{k},2}^+ s_{\mathrm{k},2}^- \rangle \tag{20}$$

where $\mathcal{N}_{1(2)}$ is the number of spins of species 1(2) in the lattice. In terms of $\alpha(\alpha^\dagger)$ and $\beta(\beta^\dagger)$ Eqs. (19) and (20) can be written as

$$\langle S_1^z \rangle = 1 - \frac{1}{\mathcal{N}_1} \sum_{\mathrm{k} \in B_z} u_{\mathrm{k}}^2 \langle \alpha_{\mathrm{k}}^\dagger \alpha_{\mathrm{k}} \rangle + v_{\mathrm{k}}^2 \langle \beta_{\mathrm{k}} \beta_{\mathrm{k}}^\dagger \rangle \tag{21}$$

$$\langle S_2^z \rangle = -1 + \frac{1}{\mathcal{N}_2} \sum_{\mathrm{k} \in B_z} v_{\mathrm{k}}^2 \langle \alpha_{\mathrm{k}} \alpha_{\mathrm{k}}^\dagger \rangle + u_{\mathrm{k}}^2 \langle \beta_{\mathrm{k}}^\dagger \beta_{\mathrm{k}} \rangle \tag{22}$$

At equilibrium $\langle \alpha_{\mathrm{k}} \alpha_{\mathrm{k}}^\dagger \rangle = n_{\alpha_{\mathrm{k}}} = k_B T / \hbar\omega_\alpha(\mathbf{k})$ and similar for $\langle \beta_{\mathrm{k}}^\dagger \beta_{\mathrm{k}} \rangle = n_{\beta_{\mathrm{k}}} = k_B T / \hbar\omega_\beta(\mathbf{k})$. We note that we are dealing with the classical spin Heisenberg model and thus the thermal equilibrium distribution of the spin fluctuations rest on the classical limit. Quantum statistics is easy to consider and this will not change the qualitative results. *Discussion of frequencies.* Limiting cases:

- $J_{12} = 0$. Two non-coupled ferromagnetic lattices. In this case the non-diagonal terms in matrix Eq. (12) are zero and thus the matrix is diagonal, so that $\omega_\alpha(\mathbf{k}) = \Omega_1(\mathbf{k}) = \frac{\gamma}{\mu_1} m_1 \left(J_1(0) - J_1(\mathbf{k})\right)$ and $\omega_\beta(\mathbf{k}) = \Omega_2(\mathbf{k}) = \frac{\gamma}{\mu_2} m_2 \left(J_2(0) - J_2(\mathbf{k})\right)$. The eigen-vectors coefficients are then; $u_{\mathrm{k}} = \pm 1$ and $v_{\mathrm{k}} = 0$, where the sign depends on the chosen spin fluctuation gyration direction. The spins fluctuations at each sublattice are obviously not mixed. Thermal excitation of $\alpha(\beta)$ magnons only will lead to a reduction on the sublattice 1(2) but not of the sublattice 2(1). This lack of mixing between sublattices is reflected by the product $2u_{\mathrm{k}} v_{\mathrm{k}} = 0$. At $k \to 0$, both $\Omega_n(k \to 0) \sim Jk^2$ which characterises ferromagnetic spin waves. As the temperature increases excitation of ferromagnetic magnons reduces the $\langle S_i^z \rangle$.

- $J_{11} = J_{22} = 0$. In this case, $\Omega_n(\mathbf{k}) = 0$, and the matrix (12) diagonal elements are zero and both magnons α_{k} and β_{k} are degenerate with $\omega(k \to 0) \sim A_{12} k$. In the antiferromagnets magnons maximum mix of the spins fluctuations of each sublattice happens, here, $2u_{\mathrm{k}} v_{\mathrm{k}} = 1$, with $u_{\mathrm{k}}^2 = v_{\mathrm{k}}^2 = 1/2$. As the temperature increases the excitation of antiferromagnetic magnons reduces the individual lattice magnetisation $\langle S_i^z \rangle$ while it conserves the total magnetization $\langle S_{\mathrm{tot}}^z \rangle = 0$.

- For a general ferrimagnet with exchange contributions from both ferro- and antiferromagnetic exchange interactions the general solution at $k \to 0$ for the magnon frequencies written in Eq. (16) can be expressed as a combination of linear and quadratic k terms, $\Omega_\alpha(\mathbf{k}) = a_1 + b_1 k + c_1 k^2 + \mathcal{O}(k^3)$ and $\Omega_\beta(\mathbf{k}) = a_2 + b_2 k + c_2 k^2 + \mathcal{O}(k^3)$, where $a_{1(2)}$, $b_{1(2)}$ and $c_{1(2)}$ are are complicated algebraic expressions. Bearing in mind that we have considered only exchange interactions between spins in the VCA Hamiltonian (11), we obtain that one branch has the frequency $\omega = 0$ at $k = 0$. This is related to the absence of any applied or anisotropic field to break the rotational symmetry and lift the frequency of the $k = 0$ mode. The inclusion of the anisotropy field within the VCA Hamiltonian (11) will slightly increase the frequency of the magnon branches by an amount related to the anisotropy field $H_K \sim K$ and with time scales much larger ($\sim$ ns) than the studied processes ($\sim$ ps).

[1] Kaganov, M. I., Lifshitz, I. M. & Tanatarov, L. V. Relaxation between electrons and crystalline lattices. *JETP* 173 (1957).

[2] Chen, J. K., Tzou, D. Y. & Beraun, J. E. A semiclassical two-temperature model for ultrafast laser heating . *International Journal of Heat and Mass Transfer* **49**, 307–316 (2006).

[3] Bergman, A. *et al.* Magnon softening in a ferromagnetic monolayer: a first-principles spin dynamics study. *Phys. Rev. B* **81**, 144416 (2010).

[4] Hoshen, J., Berry, M. & Minser, K. Percolation and cluster structure parameters: The enhanced Hoshen-Kopelman algorithm. *Phys. Rev. E* **56**, 1455–1460 (1997).

[5] Stauffer, D. & Aharoni, A. *Introduction to Percolation Theory* (Taylor & Francis, London, 1994), 2 edn.

[6] Grassberger, P. Numerical studies of critical percolation in three dimensions. *J. Phys. A: Math. Gen.* **25**, 5867 (1992).

[7] Lorenz, C. & Ziff, R. Precise determination of the bond percolation thresholds and finite-size scaling corrections for the sc, fcc, and bcc lattices. *Phys. Rev. E* **57**, 230–236 (1998).

[8] Ostler, T. *et al.* Crystallographically amorphous ferrimagnetic alloys: Comparing a localized atomistic spin model with experiments. *Phys. Rev. B* **84**, 024407 (2011).
